# Proposed Specifiers for Conduct Disorder-Short Version (PSCD-SV): Psychometric Properties, Concurrent Correlates and Parenting Predictors

**DOI:** 10.1007/s10578-022-01335-6

**Published:** 2022-02-25

**Authors:** Laura López-Romero, Olalla Cutrín, Lorena Maneiro, Randall T. Salekin

**Affiliations:** 1 Department of Clinical Psychology and Psychobiology, Facultad de Psicología, Universidade de Santiago de Compostela, Campus Sur, 15782 Santiago de Compostela, Spain; 2 Institute of Education and Child Studies, Leiden University, Leiden, The Netherlands; 3 Department of Psychology, The University of Alabama, Tuscaloosa, USA

**Keywords:** PSCD-SV, Psychopathic traits, Conduct disorder, Parenting practices, Adolescence

## Abstract

The present study aims to further examine the four-factor model of psychopathy in adolescence with a new alternate model for the assessment of psychopathic traits and conduct disorder (CD): The Proposed Specifiers for Conduct Disorder-Short version (PSCD-SV). Data were collected in a sample of 414 adolescents (49.2% females) aged 12–15 at the first assessment who were then followed-up 2 years later. Results supported the usefulness of the PSCD-SV to assess the broader construct of psychopathy showing good psychometric properties, including adequate reliability and validity, while accounting for all its dimensions. In addition, the study showed close associations between psychopathic traits and adolescent behavioral, emotional and psychosocial maladjustment. Finally, the findings elucidated the PSCD’s connection to parental support and psychological control, and reinforced the potential role of parenting practices as predictors that can act as mechanisms of change in the development of psychopathy. Overall, current findings shed light on conceptual and developmental models of psychopathy that may have implications for assessment, diagnostic classification, prevention, and intervention.

Psychopathy or psychopathic personality has been traditionally defined as constellation of co-occurring interpersonal (e.g., deceitfulness, grandiosity), affective (e.g., callousness, lack of remorse), and behavioral dimensions (e.g., impulsivity, irresponsibility), in addition to antisocial traits and behaviors [1]. Largely based on the work developed through the Psychopathy Checklist-Revised [2], factor analytical studies have explored different models and structures. Initially, a two-factor structure, including an interpersonal-affective dimension (Factor 1) and an impulsive-antisocial dimension (Factor 2) was revealed [3]. A three-factor model was later proposed by separating Factor 1 into two components (i.e., interpersonal and affective) and dropping antisocial items from Factor 2 to form a single lifestyle component [4]. Later, the traditional two-factor model of psychopathy was reformulated as a four-factor construct reincorporating antisocial behavior with interpersonal (facet 1) and affective (facet 2) domains comprising the traditional Factor 1, and lifestyle (facet 3) and antisocial behavior (facet 4) as separate domains of traditional Factor 2 [1, 2].

During the past two decades, research on psychopathic personality has also been extended downward in age, being studied in childhood and adolescence [5, 6]. In this regard, prior research has described child psychopathy as a multifaceted construct, including interpersonal [i.e., Grandiose-Manipulative (GM)], affective [i.e., Callous-Unemotional (CU)], and behavioral/lifestyle dimensions [i.e., Daring–Impulsive (DI)] that can be observable at an early age [7], are relatively stable across time [8], and show specific meaningful correlations with cognitive, emotional, personality and psychosocial variables [9]. In line with adult literature, two-, three-, and four-factor solutions were also statistically explored at early developmental stages, with most studies conducted with diverse samples of children and adolescents supporting a three-factor structure [7, 10–13], albeit some evidence for a four-factor structure has been also provided [12, 14, 15]. Even considering a three- or a four-factor model when assessing psychopathic traits in young samples, all psychopathy dimensions have been shown to be associated with a large set of psychosocial and behavioral problems, including conduct problems, aggression, low prosocial behavior, antisocial behavior and offending [16].

Further support for considering the entire syndrome, as well as recognizing all its dimensions, has been recently provided. Different studies conducted in different samples of preschoolers, school-aged children and adolescents from several countries, found that the combination of high levels of all three psychopathy dimensions (i.e., interpersonal, affective, and behavioral/lifestyle) and conduct problems was most strongly related to child and youth behavioral and psychosocial maladjustment (e.g., conduct problems, aggression, ADHD) measured both concurrently and prospectively [17–22]. From these results, the authors conclude that the multidimensional model for child and adolescent psychopathy, in combination with concurrent conduct problems, seems to be more effective for predicting behavioral problems than considering only CU traits and, therefore, it may offer greater utility to researchers and clinicians for both prediction and specification of conduct disorder (CD) [16].

Although there seems to be enough support for considering psychopathy as a multidimensional construct, additional efforts for examining the broader construct as well as its component parts are still needed in order to better account for their predictive value when identifying a high-risk profile of problematic youths [23]. Although there are measures for child psychopathy, no measures exist to tap all components at once while also including CD criteria. To this end, the Proposed Specifiers for Conduct Disorder (PSCD) [6] has been developed as a measure of the broader psychopathy construct from early childhood to late adolescence, including CD. The PSCD is composed of 24 items, addressing four dimensions (specifiers) that include the interpersonal (i.e., GM), affective (i.e., CU) and lifestyle (i.e., DI) dimensions of psychopathic personality, in addition to CD. Item selection for the PSCD was performed using both rational and empirical criteria and according to three main premises: (1) to provide a measure of the three-factor model of psychopathic personality plus CD in a way that closely resembles how it is often conceptualized in adolescence and adulthood [2], but also including only those traits with an empirical and/or theoretical support for being identifiable at early developmental stages [7, 23]; (2) to increase the homogeneity within scales with item selection focused on content representativeness and item harmonization [9]. This latter point was to only include items that did not contradict one another (e.g., impulsive vs. planful/manipulative); and, (3) to include the four criteria for CD, plus one oppositional defiant disorder (ODD) item.

There are initial signs that the PSCD is a promising measure [12, 15]. For instance, the PSCD was preliminary validated in a sample of 2,229 preschool children, with information provided by parents [12]. Confirmatory factor analyses supported both three- and four-factor structures. The validity of the PSCD was also supported by convergent and divergent associations with an alternative measure of psychopathic traits as well as by the expected relations with fearlessness, conduct problems, reactive and proactive aggression, attention-deficit hyperactivity disorder, ODD, and social competence skills. In a second study, the PSCD self-report version was examined in two samples of Portuguese youths [24], including a community sample of boys and girls (n = 648), and a sample of boys from forensic settings (n = 258). Results overall supported the PSCD as representing both a general factor and four specific factors (GM, CU, DI, CD), and provided evidence for reliability, construct, and temporal validity. Finally, the psychometric properties of the self-reported version of the PSCD were also examined in a community sample of 1,683 Chinese adolescents [15]. Again, the results supported the four-factor structure of the PSCD, which better fitted in a bifactor structure. The authors also proposed a short version (PSCD-SV) including 13 items with substantial item reliability, which provided further support for a four-factor interrelated model, being in line with the initial conceptualization of the PSCD. Also, the short version provided an even better fit to the data and identified a significantly higher proportion of youths with elevated psychopathic traits, suggesting that the short version slightly outperformed the standard version, at least at the psychometric level. Finally, both standard and short versions exhibited the expected relations with other psychopathy measures, anxiety and depression, and aggression.

Overall, results obtained to date with the PSCD suggest that youth psychopathy is a multifaceted construct that can be modeled with CD as originally proposed with important relations with external theoretically meaningful variables [9, 23]. Although the PSCD seems a promising measure, much more research is needed given that very few studies exist on the PSCD. Research that continues to evaluate the psychometric properties of the PSCD, especially research that expands on the examination of external correlates and potential developmental precursors, will help to build the knowledge base for this relatively new, and only minimally investigated, PSCD measure.

## Developmental Precursors of Psychopathic Personality: The Role of Parenting

The relevance of psychopathic traits for long-lasting behavioral and psychosocial problems makes it a necessary consideration to better understanding their development by examining some pertinent precursors. That is, although biological (i.e., genetics) [25] and temperamental factors (i.e., fearlessness) [26, 27], have been extensively highlighted as early underpinnings of psychopathic personality, from a developmental psychopathology approach environmental influences (e.g., parenting practices) should also be considered as potential markers of psychopathic traits [28] as they may shed light on the mechanisms of change that may influence their later development across lifespan [9]. However, at the child level, studies on parenting practices appears to mainly be focused on only one component of psychopathy, namely CU traits. And, even then, the parenting practices are only moderately investigated. This makes it difficult to know how the broader concept and its individual components may related to parenting practice and underscores the need for multidimensional measures like the PSCD to fill this void.

As was consistently observed for behavioral problems [29–33], dysfunctional parenting practices, including inconsistent, harsh and coercive practices in addition to low warmth, support, affection and acceptance, emerge as relevant factors in predicting changes in psychopathic traits across childhood and adolescence [9, 34, 35], for comprehensive reviews]. Parental warmth, responsiveness or acceptance has been used to refer to the support dimension of parenting (parental support, from now on) that represents parenting behaviors that make the child feel loved, accepted and approved [30]. Results from previous research showed that an infrequent use of practices based on warmth, acceptance and involvement was related with higher levels of CU traits [36–38]. Moreover, adolescents high on CU traits reported low parent–child involvement [39, 40], low monitoring [39, 41], deficient parent–child communication patterns [40] as well as low autonomy transfer, and high levels of harsh parenting [42, 43]. Furthermore, overall psychopathic and more specific CU traits were found as moderators in the relationship between parenting practices and adolescent outcomes and behaviors [44–48].

As noted, since current conceptualizations of youth psychopathy encourage a multidimensional approach to the construct [6, 9], new studies aimed at disentangling the effect of different parenting dimensions on each psychopathy dimension will help to better understand the environmental contribution to their development across time. Previous studies in this regard did not show significant associations between harsh and inconsistent parenting and any of the four psychopathic facets assessed by means of the PCL-YV [49]. Also, the study carried out by Chinchilla and Kosson [44] did not find a significant association between parental warmth and the interpersonal and affective facets of psychopathy; however, they found a significant association with the total PCL-YV. Most of these studies did not longitudinally analyze the relationship between parenting and psychopathic traits, which prevents the consideration of parenting as a precursor of psychopathy. To disentangle the effects between parental behavior and psychopathic traits over time, Salihovic et al. [50] conducted a longitudinal study in Sweden. In this study, negative parental practices, such as parent’s negative reactions to disclosure, and positive parenting reactions, such as attempted understanding, predicted change in adolescent psychopathic traits over time as revealed by a cross-lagged panel model. More recently, Backman et al. [51] observed in a large sample of offending adolescents that adolescent self-reported parental warmth was associated with lower psychopathic traits, whereas high hostility was predictive of higher psychopathic traits. However, they used a global score for psychopathic traits, which did not allow to explore which dimension(s) particularly accounted for those associations. Therefore, further research is needed in order to elucidate which parenting factors and processes are specifically linked to overall psychopathic personality and each particular dimension, promoting either stability or change, and providing new insight for theoretical understanding, prevention targets, risk assessment, and intervention designs.

## This Study

The present study was developed with the primary purpose of further examining the psychometric properties of the PSCD-SV including its factor structure, reliability, and convergent associations of the PSCD-SV scales and Total scores with a wide range of behavioral, emotional and psychosocial variables traditionally shown to be related with the psychopathy construct, but barely examined when using the PSCD thus far (e.g., externalizing and internalizing problems, and antisocial behavior). This study builds on past research with the PSCD with the examination of the parenting variables which offers a new and needed area of investigation for both psychometric investigation purposes but also potential etiological theory development. The parental component of the study is measured at two time points providing a longitudinal component to the investigation. In addition, this study focused on the short version of the PSCD, given that there is a need for brief measures of psychopathy for research purposes [12]. The usefulness and increasing need of short scales for measuring personality traits has been widely recognized [52], since they facilitate data collection (e.g., screening studies, large-scale surveys) in a cost-effective manner [15]. In sum, the psychometric properties of the PSCD-SV, including factor structure and internal consistency, were preliminary examined in a sample of adolescents, followed by convergent validity and an examination of parental variables in a longitudinal design. Although the PSCD has some preliminary data to support its use, we expected in the current study that it would show adequate psychometric properties, before drawing developmental conclusions on the parenting variables.

The following hypotheses were proposed: (1) a better fit for the four-factor model in regard to the internal structure of the PSCD is expected, which resembles the four dimensions encompassed in the construct of psychopathy (i.e., GM, CU, DI, and CD); (2) the PSCD-SV was expected to show good internal consistency for a short scale; (3) these psychopathic traits were also expected to show positive associations with behavioral, emotional, and psychosocial problems, including antisocial behavior, as well as positive relationships with parenting practices such as psychological control and negative associations with parental support, backing the convergent validity of the scale; and (4) parenting practices were expected to longitudinally and distinctively predict the four-psychopathy dimensions in a two-year period. Specifically, it was expected that both psychological control and parental support would strongly predict the affective facet of psychopathy (i.e., CU) as well as CD, whilst significant differential associations are also expected for GM and DI.

## Methods

### Participants

The sample used in the current study is part of a three-year longitudinal study which was conducted in the Autonomous Community of Galicia (NW Spain) between 2017 and 2019. The initial sample included a total of 642 adolescents in the first grade of compulsory secondary education [1° ESO] from 11 state secondary schools. Participants in T1 ranged in age from 12 to 15 (*M* = 12.49; *SD* = 0.67), and 45.4% were females. The second wave that took place one-year after the first assessment (T2) included a total of 625 adolescents aged 13–17 (*M* = 13.43; *SD* = 0.68), of which 47.5% were females. Finally, a total of 627 adolescents took part in the third wave which was carried out 2 years after the first assessment (T3). Participants in T3 aged 14–18 (*M* = 14.42; *SD* = 0.68), 48.6% females, and were involved in the third grade of compulsory secondary education [3° ESO]. It should be noted that second and third waves included not only participants assessed at the first wave but also new participants that met the criteria for their inclusion in the study. For the purposes of the current study, only adolescents who were assessed at T1 and followed-up at T3 were included in the analysis. This gave rise to a total sample of 414 adolescents aged 12–15 at the first assessment (*M* = 12.32; *SD* = 0.50; 49.2% females), which was followed up 2 years later when participants were in third grade (*M* = 14.18; *SD* = 0.40). Most of participants lived with both parents at T1 (84.7%), whereas 12.1% lived only with their mother, 1.9% lived only with their father, and 1.2% lived with other relatives. More than 90% of the sample were Galician, white, and came from middle and low-middle socio-economic backgrounds.

### Measures

#### Psychopathic Traits (T3)

The Proposed Specifiers for Conduct Disorder-Short Version (PSCD-SV) [6, 15] was used for the self-reported assessment of psychopathic traits. The short version of the PSCD was developed by retaining 13 items from the original PSCD with substantial item reliability for the three psychopathy dimensions: GM (3 items, e.g., “Lying is easy for me”), CU (3 items, e.g., “I don’t waste time thinking about how I may have hurt others”), and DI (3 items, e.g., “I get a thrill out of doing risky things”); and the four CD subtype items (4 items, e.g., “I have stolen things”) (15)]. The PSCD-SV was rated by participants using a 3-point scale (0 = *not true*, 1 = *sometimes true*, 2 = *true*). Additional information about the items, factor loadings and reliability of the factors are presented in Tables 1 and 2 and the “Results” section.

**Table 1:** Descriptive statistics for main study variables

	Mean	SD	Range^a^	
			Min	Max
T1 variables				
Parenting				
Support	2.47	0.58	0.00	3.00
Psychological control	0.90	0.65	0.00	3.00
Antisocial behavior	0.48	0.97	0.00	7.75
T3 variables				
PSCD-SV				
GM	0.37	0.36	0.00	2.00
CU	0.23	0.33	0.00	1.67
DI	0.82	0.56	0.00	2.00
CD	0.19	0.32	0.00	1.75
Total score	0.35	0.27	0.00	1.62
SDQ				
Conduct problems	0.32	0.30	0.00	1.40
Emotional problems	0.60	0.48	0.00	2.00
Hyperactivity	0.85	0.48	0.00	2.00
Peer problems	0.28	0.29	0.00	1.40
Prosocial behavior	1.58	0.34	0.40	2.00
ABQ				
Aggressive behavior	0.07	0.20	0.00	1.83
Rule-breaking	0.21	0.33	0.00	3.00
Theft	0.09	0.23	0.00	2.17
Vandalism	0.07	0.20	0.00	1.67
Parenting				
Support	2.21	0.64	0.00	3.00
Psychological control	1.13	0.71	0.00	2.88

*PSCD-SV* Proposed Specifiers for Conduct Disorder-Short Version, *GM*  grandiose-manipulative, *CU* callous-unemotional, *DI* daring-impulsive, *CD* conduct disorder, *SDQ* Strengths and Difficulties Questionnaire, *ABQ* Antisocial Behavior Questionnaire

^a^Range of mean scores for each analyzed variable in the current sample

**Table 2:** Item loadings for the four-factor solution (CFA) of the PSCD-Short Version

	GM	CU	DI	CD
Item				
1. Lying is easy for me	0.77			
2. I take advantage of others	0.78			
3. I am a good storyteller	0.42			
4. I don’t waste time thinking about how others feel		0.61		
5. When people are happy or upset I don’t seem to care		0.59		
6. I like it when others are afraid of me		0.71		
7. I like a lot of change or adventure			0.40	
8. I get a thrill out of doing risky things			0.63	
9. I feel like I need a lot of stimulation			0.71	
10. I have stolen things				0.85
11. I have engaged in physical aggression against animals or people				0.59
12. I have destroyed property				0.72
13. Some people say I break a lot of rules				0.67
Factor loadings on total score	0.85	0.66	0.66	0.94

*PSCD* Proposed Specifiers for Conduct Disorders, *CFA* confirmatory factor analysis, *GM* grandiose/deceitful, *CU* callous-unemotional, *DI* daring/impulsive, *CD* conduct disorder. All factor loadings were statistically significant at *p* < 0.001

#### Parental support (T1–T3)

The perception of parental warmth, responsiveness, and closeness to parents was assessed by means of the self-reported Affection and Communication scale, developed and validated in community Spanish adolescents as part of a larger parenting questionnaire [53], and used in previous studies with the current sample [54]. The scale is composed of 8 items (e.g., “You feel supported and understood”) scored in a 4-point scale ranging from 0 (*never*) to 3 (*always*). The internal consistency of the scale (i.e., Cronbach’s alpha) for the total sample was 0.89 in the first wave (Mean Inter-item Correlations [MIC] = 0.70) and 0.91 in the third wave of study (MIC = 0.72).

#### Psychological Control (T1–T3)

Also included in the parenting questionnaire developed and validated by Oliva et al. [53], the self-reported Psychological Control scale, was used for the assessment of parental psychological control, defined as the use of psychological coercion and emotional manipulation through which parents seek to control or manage their children's behavior. The scale was composed of 8 items (e.g., “Your parents make you feel guilty when you don't do what they want”) which ranged from 0 (*never*) to 3 (*always*). The Cronbach’s alpha for the total sample was 0.86 in the first wave (MIC = 0.62) and 0.89 in the third wave (MIC = 0.66).

#### Psychosocial Adjustment (T3)

The Spanish self-reported version of the Strengths and Difficulties Questionnaire (SDQ) [55, 56] was used for the assessment of the adolescent psychosocial functioning. The SDQ is composed of 25 items intended to assess five different psychosocial domains (each with five items): Emotional symptoms (e.g., “I am often unhappy, depressed or tearful”, α = 0.73, MIC = 0.50), Conduct problems (e.g., “I fight a lot. I can make other people do what I want”, α = 0.49, MIC = 0.28), Hyperactivity (e.g., “I am easily distracted, I find it difficult to concentrate”, α = 0.69, MIC = 0.45), Peer problems (e.g., “Other children or young people pick on me or bully me”, α = 0.51, MIC = 0.29), and Prosocial behavior (e.g., “I am helpful if someone is hurt, upset or feeling ill”, α = 0.63, MIC = 0.39). The response format ranged in a 3-point scale from 1 (*not true*) to 3 (*certainly true*).

#### Antisocial Behavior (T1–T3)

Problematic behavior was assessed by means of the Antisocial Behavior Questionnaire (ABQ) [57]. Four subscales (6 items each) were included in this study (T3): aggression (e.g., “Fighting and hitting someone”; α = 0.72, MIC = 0.50), rule-breaking behavior (e.g., “Spending the night out without permission”; α = 0.63, MIC = 0.38), theft (e.g., “Taking something from class without permission with the intention of stealing it”; α = 0.63, MIC = 0.38), and vandalism (e.g., “Setting fire to something: a bin, table, car, etc.”; α = 0.63, MIC = 0.38). A composite mean score was created with the scales measured in T1 to be included as covariate in the SEM models (α = 0.77, MIC = 0.59). All the items were scored in a 4-point scale which ranged from 0 (*never*) to 3 (*very often*).

### Procedure

The procedure followed throughout the investigation was in compliance with the standards of the University Ethics Committee and the Declaration of Helsinki. Following a convenience sampling approach, the heads of 24 secondary schools were initially contacted by phone to present the study and ask for potential collaboration. Schools located in the four provinces of the Autonomous Community, and representative of this area, were contacted. A brief report with more detailed information about the research project and the goals of the study were sent by mail to all those interested in taking part in. Specifically, the main goal of this longitudinal study was to delve into the role that certain parenting practices have on adolescent development, as well as the interactional effect with other factors such as peers or personality traits. Hence, to analyze the psychosocial development from early adolescence, the target population of study encompassed all students involved in the first degree of secondary education at the beginning of the project. After the first contact, a total of 11 secondary schools agreed to participate in the longitudinal study. Qualified psychologists from the research group visited all the schools to explain the procedures and solve any doubts that may arise regarding the prospective study. Parental consent was requested each year before the beginning of data collection and, subsequently, adolescent assent was obtained during questionnaire implementations. Given that informed consent was annually requested, no restrictions regarding new participations were made for their inclusion in the study. Data collection was conducted by qualified research assistants, who visited the centers during school hours in order to provide proper instructions to the adolescents and monitor the process. Adolescents filled out the questionnaires in group sessions of approximately 50 min in classroom. Adolescent participation was voluntary, and confidentiality was guaranteed following the ethical criteria. Personal but anonymous codes were used to link questionnaires’ information from different waves of study. A total of three waves of data collection were carried out during a three-year period, with intervals of approximately 12 months between follow-ups.

### Data Analysis

Descriptive statistics for main study variables were first examined in SPSS 25. Second, in order to replicate the factorial structure of the short version of the PSCD proposed by Luo et al. [15], a Confirmatory Factor Analysis (CFA) was conducted in Mplus 7.4 [58], with robust weighted least squares used as estimator (WLSMV). A four-factor interrelated and a four-factor superordinate model were specified, including the 13 items as observed variables and psychopathy as a second order factor. Model fit was assessed using root-mean-square error of approximation (RMSEA), comparative fit index (CFI), and the Tucker–Lewis index (TLI). According to Hu and Bentler [59] suggestions, RMSEA values lower or equal to 0.05, and TLI and CFI values of 0.95 or higher were considered indicators of good model fit, whereas a RMSEA smaller than 0.08, and TLI and CFI larger than 0.90 indicated adequate model fit. Internal consistency of the PSCD-SV total score was computed with Cronbach’s alpha (α). However, given the dependence of alpha on the number of items in a scale, as well as other concerns about its reliance (e.g., the normal distribution of items) [60], mean inter-item correlation (MIC) was computed as a more appropriate indicator of the internal consistency for the PSCD-SV subscales (i.e., 3 items per subscale), with values ranging from 0.15 to 0.50 being considered adequate [61]. Concurrent associations of the PSCD factors and Total score with external variables measuring behavioural, emotional and psychosocial problems, antisocial behaviour and parenting practices were examined by computing zero-order correlations in SPSS 25. Partial correlations controlling for age and gender were computed for PSCD Total. For each PSCD factor, we also computed partial correlations controlling for the effect of gender, age and the other three PSCD factors. In this way, we were able to examine the differential associations of each PSCD factor with external criteria, above and beyond the shared variance among dimensions. To counteract the issue of multiple testing, Bonferroni’s correction was applied, and the threshold levels of significance were settled at 0.005 (11 variables). Finally, a Structural Equation Model (SEM) was computed in Mplus 7.4 using WLSMV as estimator, to analyze the influence of parenting practices in early adolescence on psychopathic traits 2 years later, including both parental support and parental psychological control as latent variables predicting the four latent psychopathic factors. SEM controlled for age and gender of the participants, people they lived with (coexistence), and antisocial behavior in T1. All models adjusted statistically for the school-level clustering of data and used listwise to manage missing data.

## Results

### The PSCD-SV: Psychometric Properties

**Fig. 1:**
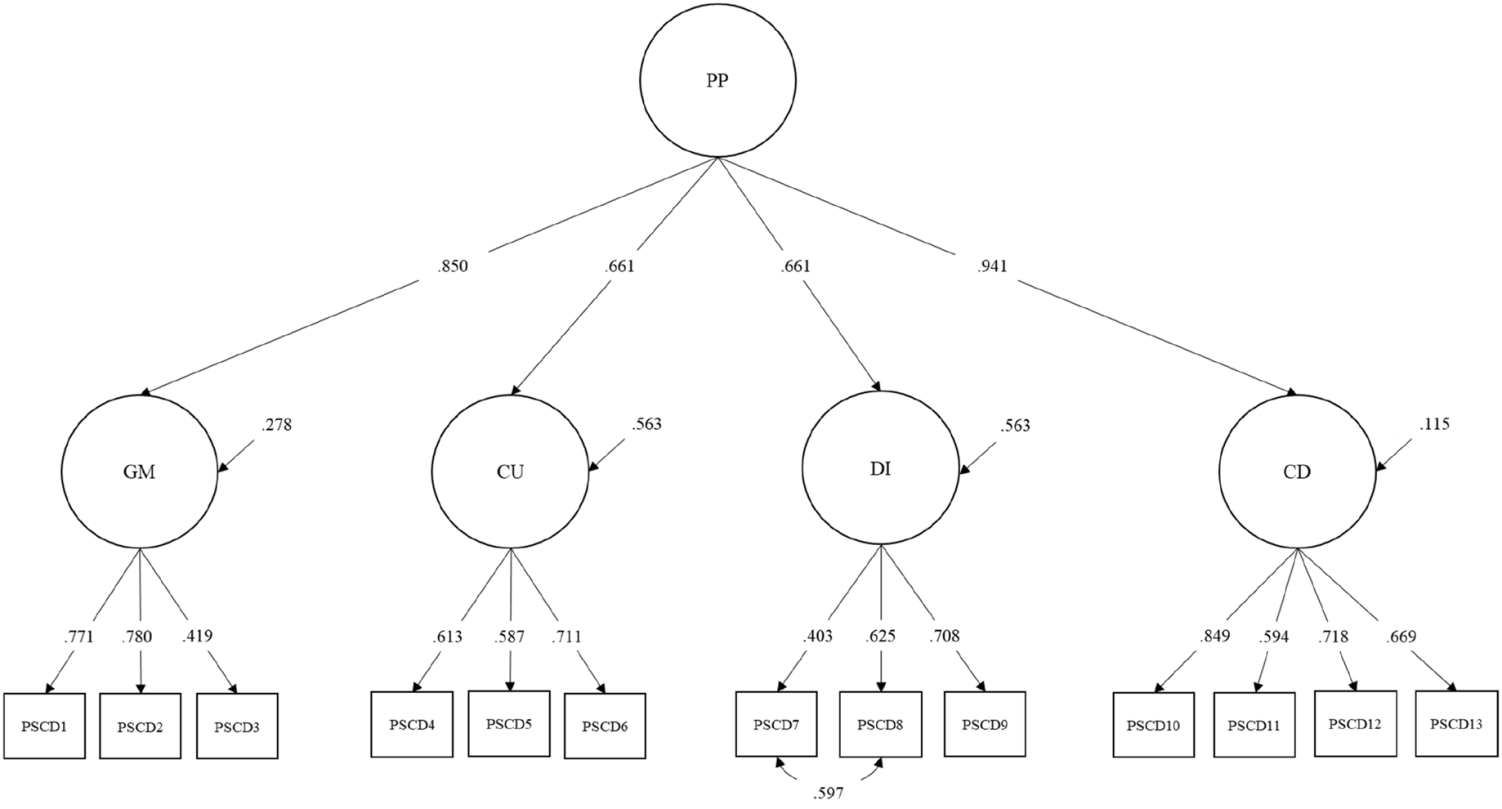
Standardized model parameters for the four-factor superordinate model of the Proposed Specifiers for Conduct Disorder-Short Version (PSCD-SV). *PP* Psychopathic Personality, *GM* grandiose/deceitful, *CU* callous-unemotional, *DI* daring/impulsive, *CD* conduct disorder, *PSCD* Proposed Specifiers for Conduct Disorder. Fit indices and factor loadings were equivalent between the superordinate and the four-factor interrelated models. In the four-factor interrelated, all PSCD-SV dimensions were significantly correlated (*p* ˂ 0.001): GM–CU = 0.60; GM–DI = 0.57; GM–CD = 0.78; CU–DI = 0.36; CU–CD = 0.63; DI–CD = 0.65

The four-factor model of the PSCD-SV showed an acceptable-to-good model fit, χ^2^ (60) = 100.92, *p* ˂ 0.001, CFI = 0.94, TLI = 0.92, RMSEA = 0.05 [0.04, 0.07]. The four-factor model was better than the one-factor model, χ^2^ (65) = 313.14, *p* ˂ 0.001, CFI = 0.78, TLI = 0.73, RMSEA = 0.10, and the three-factor model χ^2^ (24) = 74.11, *p* ˂ 0.001, CFI = 0.93, TLI = 0.89, RMSEA = 0.07. Modification indices substantiated a residual covariance between items 15 and 14 as representing an acutely misspecified parameter in the model. After including this new parameter, a good model fit was observed for the four-factor model, χ^2^ (60) = 100.92, *p* ˂ 0.001; CFI = 0.96, TLI = 0.95, RMSEA = 0.04 [0.03, 0.05]. Equal model fit indices were obtained for the two alternative four-factor interrelated and four-factor superordinate models (see Fig. 1 for a representation of the four-factor superordinate model). As can be observed in Table 2, all 13 items loaded significantly on the expected PSCD factor and the latent global construct, with all item and factor loadings being above 0.40.

In terms of internal consistency, Cronbach’s alpha was 0.73 for the PSCD Total score. Given the very low level of number of items for the subscales, we utilized the MIC to further examine scale homogeneity. All MIC values were indicative of an adequate internal consistency for all the PSCD factors (MIC = 0.30, 0.28, 0.47, 0.43 for GM, CU, DI, and CD respectively). The MIC for the Total score also showed good homogeneity (MIC = 0.35). Significant correlations (*p* < 0.001) were observed between the PSCD Total score and the four PSCD factors (r’s = 0.69^GM^; 0.55^CU^; 0.73^DI^; 0.74^CD^).

### Concurrent Associations Between Psychopathic Traits and External Criteria

As displayed in Table 3, the PSCD Total score was significantly correlated with all analyzed variables. At the zero-order level, all PSCD factors showed significant correlations with conduct problems and all facets of antisocial behavior. DI and CD traits were significantly correlated with emotional problems and hyperactivity, which was also related with the GM factor. All GM, CU and CD factors were negatively correlated with prosocial behavior, whilst only CU traits showed a significant correlation with peer problems. With respect to parenting practices, GM, CU, and CD factors were negatively correlated with parental support, whilst only GM and CD significantly correlated with psychological control.

**Table 3:** Concurrent (T3) zero-order and partial correlations (controlling for the other three PSCD subscales), between the PSCD subscales and total score and external criteria

	PSCD				
	GM^a^	CU^a^	DI^a^	CD^a^	Total score
SDQ					
Conduct problems	0.48* (0.30*)	0.25* (0.07)	0.33* (0.17*)	0.49* (0.31*)	0.56*
Emotional problems	0.13 (0.07)	0.07 (0.02)	0.11* (0.05)	0.14* (0.07)	0.17*
Hyperactivity	0.32* (0.17*)	0.10 (− 0.05)	0.36* (0.26*)	0.33* (0.18*)	0.42*
Peer problems	0.12 (0.08)	0.16* (0.13)	0.04 (0.00)	0.09 (0.01)	0.14*
Prosocial behavior	− 0.24* (− 0.14*)	− 0.31* (− 0.24*)	− 0.01 (0.12)	− 0.25* (− 0.13)	− 0.26*
ABQ					
Aggressive behavior	0.20* (− 0.03)	0.24* (0.12)	0.15* (0.01)	0.47* (0.41*)	0.38*
Rule-breaking	0.26* (0.03)	0.17* (0.03)	0.32* (0.20*)	0.46* (0.35*)	0.45*
Theft	0.28* (− 0.02)	0.24* (0.10)	0.27* (0.12)	0.57* (0.48*)	0.50*
Vandalism	0.21* (0.01)	0.26* (0.12)	0.24* (0.13)	0.38* (0.30*)	0.39*
Parenting					
Support	− 0.24* (− 0.14*)	− 0.15* (− 0.05)	− 0.11 (− 0.01)	− 0.25* (− 0.15*)	− 0.26*
Psychological control	0.24* (0.15*)	0.09 (0.01)	0.11 (0.12)	0.24* (0.14*)	0.24*

*PSCD* Proposed Specifiers for Conduct Disorder, *GM* grandiose-manipulative, *CU* callous-unemotional, *DI* daring-impulsive, *CD* conduct disorder, *SDQ* Strengths and Difficulties Questionnaire, *ABQ* Antisocial Behavior Questionnaire

^a^Partial correlations controlling for the other three PSCD factors

^*^Significant *p* value after applying Bonferroni correction (*p* < 0.005)

When controlling for the effect of the other psychopathic traits, the GM factor remained significantly correlated with conduct problems, hyperactivity, prosocial behavior, and parental support (both inversely) and psychological control (see Table 3). The CU factor, in contrast, only showed a unique significant (inverse) correlation with prosocial behavior. The DI factor remained significantly correlated with conduct problems, hyperactivity, and rule breaking. Finally, the CD factor remained significantly correlated with conduct problems, hyperactivity, all facets of antisocial behavior, and both parenting practices.

### Examining the PSCD Construct Validity via Longitudinal Effects of Parenting on Psychopathic Traits

**Fig. 2:**
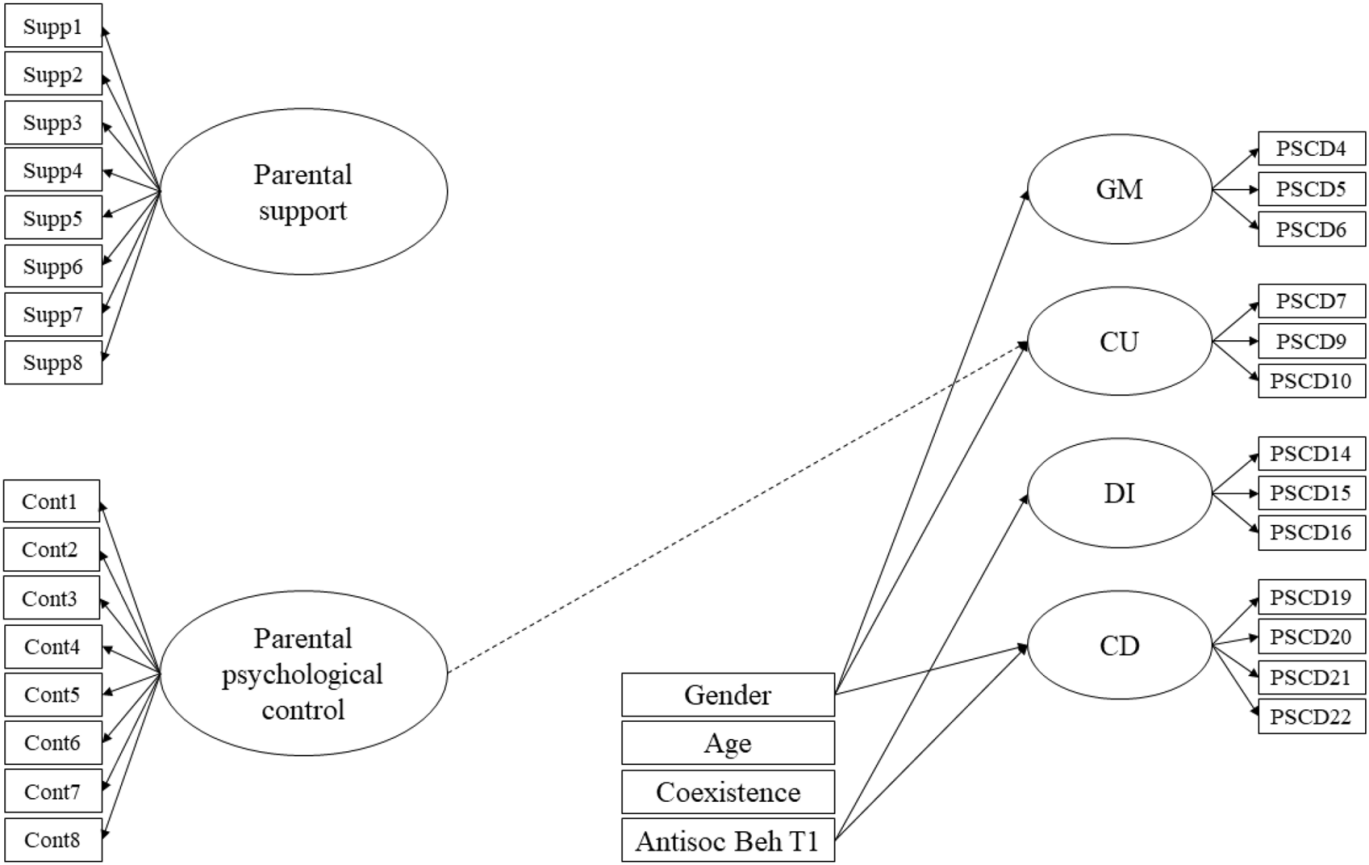
Significant results of the SEM parenting model predicting psychopathic traits. Dotted line represents significant relationships in non-standardized results. *GM* Grandiose/deceitful, *CU* Callous-unemotional, *DI* Daring/Impulsive, *CD* conduct disorder

A SEM model was conducted to predict PSCD psychopathic traits two years following the initial assessment, including both parental support and psychological control as predictors. For methodological issues, the SEM model was based on the four-factor interrelated model of the PSCD-SV, allowing to test the unique association between parenting predictors and later psychopathic traits. This model, statistically equivalent than the four-factor superordinate model, provided a more parsimonious solution to be accommodated in SEM. In order to preliminary test the independent contribution of each parenting practice, two independent models were also tested. One included parental support as independent predictor of later psychopathic traits, χ^2^ (241) = 264.41, *p* = 0.144, CFI = 0.96, TLI = 0.95, RMSEA = 0.02 [0.00, 0.03], showing that support significantly predicted lower levels of GM (*β* = − 0.27), CU (*β* = − 0.27), and CD (*β* = − 0.14). An alternative model included psychological control as T1 predictor χ^2^ (242) = 269.63, *p* = 0.107, CFI = 0.96, TLI = 0.95, RMSEA = 0.02 [0.00, 0.03], showing that it significantly predicted higher levels of CU (*β* = 0.28) and CD (*β* = 0.18) (further details are available upon request). The integrated model, including both support and control, exhibited an adequate fit, χ^2^ (452) = 486.14, *p* = 0.129, CFI = 0.97, TLI = 0.96, RMSEA = 0.01 [0.00, 0.02]. with all psychopathic traits being significantly correlated (*r*’s = 0.72^GM−CU^; 0.59^GM−DI^; 0.75^GM−CD^; 0.41^CU−DI^; 0.60^CU−CD^; 0.66^DI−CD^; *p*s < 0.001). Table 4 displays the unstandardized and standardized results of this model, as well as the explained variance. Gender (being male) significantly predicted GM, CU, and CD, and antisocial behavior at T1 significantly predicted higher levels of DI and CD. However, including both parenting practices (*r* = − 0.58, *p* < 0.001) did not reveal a significant effect on later psychopathic traits, as observed for the non-significant standardized effects of parenting practices on psychopathic traits. Only the unstandardized effect of psychological control on CU was statistically significant (Fig. 2).

**Table 4:** Results of the SEM parenting model (T1) predicting psychopathic traits (T3)

	GM		CU		DI		CD	
	Beta (*SE*)	β	Beta (*SE*)	β	Beta (*SE*)	β	Beta (*SE*)	β
Gender	− 0.25 (0.10)**	− 0.18**	− 0.41 (0.09)***	− 0.35***	− 0.02 (0.09)	− 0.03	− 0.54 (0.08)***	− 0.32***
Age	0.02 (0.08)	0.02	− 0.10 (0.13)	− 0.08	− 0.05 (0.07)	− 0.06	0.07 (0.08)	0.04
Coexistence	− 0.17 (0.11)	− 0.10	0.12 (0.11)	0.09	− 0.05 (0.05)	− 0.06	0.10 (0.10)	0.05
Antisocial behavior	0.05 (0.06)	0.07	0.02 (0.04)	0.04	0.10 (0.02)***	0.24***	0.14 (0.04)***	0.16***
Parental support	− 0.39 (0.20)	− 0.27	− 0.23 (0.14)	− 0.19	− 0.08 (0.08)	− 0.09	− 0.14 (0.14)	− 0.08
Psychological control	0.01 (0.15)	0.01	0.16 (0.08)*	0.18	− 0.02 (0.07)	− 0.04	0.18 (0.10)	0.14
R^2^	0.15		0.26		0.08		0.23	

*GM* grandiose-manipulative, *CU* callous-unemotional, *DI* daring–impulsive, *CD* conduct disorder

The independent variables were measured at T1 and the dependent variables were measured at T3. Gender was coded 0—male, 1—female. Factor loadings of parental support ranged from 0.60 to 0.81. Factor loadings of psychological control, from 0.57 to 0.77. After applying the listwise option to manage missing data, *n* = 373. Models included the correlations between PSCD15-PSCD14 and SUPPORT2-SUPPORT1 as modification indices

***p < 0.001; **p < 0.01; *p < 0.05

## Discussion

Psychopathic personality has shown its usefulness as a relevant predictor, and potential identifier, of more severe and pervasive forms of youth behavioral and psychosocial maladjustment, including conduct problems, aggression, antisocial behavior or low prosocial behavior. Although there is evidence to support the three- and four-factor models of psychopathy in adulthood [1], there is still a need to accurately address the multidimensional construct of psychopathy, by depicting the broader construct while addressing all its dimensions in childhood and adolescence [9, 23, 62, 63]. To this end, it is important to base our research on valid and useful measures that will help to advancing our knowledge on description, etiology, and developmental course of psychopathic personality across the lifespan. To this end, this study focused on the psychometric properties and external validity of the PSCD-SV, which was designed to assess the three dimensions of psychopathy plus CD with a short form.

### The PSCD-SV

The present study aimed to provide new evidence on the psychometric properties of the PSCD [6] and, more specifically, the self-reported short version (PSCD-SV) first examined by Luo et al. [15]. The PSCD-SV addresses interpersonal (i.e., GD), affective (i.e., CU) and behavioral/lifestyle (i.e., DI) dimensions of psychopathy, along with the four key CD symptom categories of CD and one ODD criteria [9, 23, 64]. According to the original proposal [9]; see also [15], the four-factor structure of the PSCD-SV was supported, representing the three psychopathy dimensions (GM, CU, and DI) along with CD, with an overarching latent hierarchical factor. The standardized factor loadings were overall higher than 0.60, with only four in the range of 0.40–0.60, being also supportive of the PSCD as a four-factor measure that distinctively assesses four different but interrelated dimensions of the psychopathic construct [1, 9]. Due to the low number of items per scale, we relied on the MICs as an estimate of reliability. The MICs for the total and subscale scores were good and therefore supported scale homogeneity.

### Psychopathic Traits and External Criteria: Concurrent Associations

At the zero-order level, both the total score and all the PSCD-SV factors were related with conduct problems and all facets of antisocial behavior [65], and all but one were significantly correlated with hyperactive behaviors (except CU traits) and—inversely—with prosocial behavior (except DI). Only CU traits showed a significant correlation with peer problems [66]. In regards to parenting, GM, CD and the Total score showed significant correlations with both support (inverse) and psychological control, whilst CU traits were negatively correlated with parental support, similar to what has been found in past research [35, 37, 40, 67]. Of note, there was a positive correlation between the Total score and emotional problems, which was essentially driven by DI and CD traits. The co-occurrence between anxiety and CD symptoms have been previously shown in some studies in childhood and adolescence [68], and may partially explain this result. Also, even considering that individuals with psychopathic traits have been traditionally defined as low anxious [69], anxiety and other related emotional problems have been considered as differential indicators of the primary (i.e., low anxious) and secondary variants (i.e., high anxious) of psychopathy, or at least component parts of psychopathy [70], a result that should be further explored in future research.

These results tend to be substantially weaker when controlling for other PSCD-SV factors, reinforcing the assumption that psychopathy dimensions depend at least in part on each other in their relation to external correlates [7]. In this regard, all psychopathic dimensions but one (i.e., CU traits) remained significantly correlated with conduct problems and hyperactivity. Significant correlations with prosocial behavior held for GM and CU traits. Nevertheless, with regard to antisocial behavior, current results suggest that the CD factor primarily accounted for most of the correlations between the PSCD factors and external criteria that are antisocial in nature. This possibility has been an issue in some conceptions of the psychopathic construct, in which conduct problems, antisocial behavior, or CD, have been treated as correlates of psychopathic personality instead as fundamental features [71]. However, evidence for the key role of antisocial behaviors in understanding and assessing the psychopathy construct is extensive [1, 9, 72–75]. Moreover, several studies have shown that psychopathic traits are important for predicting several problematic outcomes, even after controlling for severity and the onset of conduct problems [76], or in the absence of concurrent conduct problems [77, 78].

Results from partial correlations also contrast with a large body of research based on CU traits as the potential hallmark of the construct of psychopathy, or for being considered as an identifier of a specific group of problematic children showing most serious patterns of conduct problems [79]. Also, once controlling for the effect of the other PSCD-SV psychopathic traits, CU traits only held significantly correlated with prosocial behavior [80], but not with conduct problems, suggesting that when examining CU traits, the effect of the other psychopathy factors should be also taken into account [7, 9]. In sum, regardless of whether using basic correlations or partial correlations, the correlations offer support for the validity of the PSCD and the multidimensionality of the psychopathy construct. Yet, results from partial correlations should be interpreted with caution since partialling may change variables, being difficult to know “what construct an independent variable represents once the variance shared with other independent variables is removed.” [81], p. 329]. Considering psychopathic personality as a constellation of co-occurring interpersonal, affective, and behavioural/lifestyle traits [1, 7, 10, 16], results based on their global and shared contribution should be, therefore, of primary interest.

### PSCD-SV and Parenting Practices

Results from correlations showed a concurrent link between psychopathic traits as measured by the PSCD-SV and parenting practices, reinforcing some results observed in previous research [82, 83]. Overall, these findings lend further support to the validity of the PSCD-SV. However, the role of parenting practices could be understood beyond the concurrent association with psychopathic traits and, therefore, should be examined from a developmental perspective that may lead to better understanding of the potential mechanisms of change in psychopathy development or single component development. As was observed in the present study, parental support, defined as the perception of parental warmth, responsiveness, and closeness to parents, negatively predicted GM and CU traits 2 years later. Also, psychological control, based on psychological coercion and emotional manipulation, significantly predicted later CU traits and CD. These findings largely converge with previous research supporting the association between psychopathic traits—and more specifically CU traits—and an infrequent use of practices based on warmth, acceptance and involvement [36, 84], as well as deficient parent–child communication patterns [40], low autonomy transfer, and high harsh parenting [43]. However, it should be noted that the effect of both parenting practices was no longer significant when they were included together in the model, with just a marginal association between psychological control and CU traits. This points out the importance of taking into account the shared effects between different parenting practices when testing their influence on child’s development, and raises the need of further examining the role of parenting in the development of psychopathic traits.

The current results from the PSCD-SV, as a wider set of psychopathic traits and CD, may add to the literature by including the full array of psychopathic traits rather than only focusing in on one of them (i.e., CU traits). Therefore, results showed that not only CU traits, but also GM, could be affected by low parental support, warmth and affection, even after accounting for initial levels of antisocial behavior [35]. Also, the inclusion of psychological control as a predictor variable constitutes a novelty since it has rarely been addressed in previous research on this topic, although it has proved relevant for better understanding adolescent maladjustment [30, 33]. Interestingly, when the two parenting variables are included in the model, psychological control remained significantly related with CU traits, yet marginally, raising its importance as a potential developmental precursor of the affective component of psychopathy that should be also considered as a potential target for prevention and intervention purposes.

Notwithstanding these results, it should be noted that the pattern of relationship between parenting practices and psychopathic traits tend to be complex, with psychopathic traits involved in two-way effects with parent–child interactions [85–87], and potential additional moderators affecting those associations [26]. Thereby, it has been suggested that the possible role of psychopathic traits in conferring greater risk for later developing conduct problems and antisocial behavior, might be derived by uniquely shaping dimensions of parenting practices [88], an issue that should be further examined in future research. In this regard, the association between parenting practices and psychopathic traits should be interpreted in the context of potential gene-environment interactions [89], where heritable patterns would potentially underpin the development of psychopathic traits [90], whilst parenting practices would play a role as a potential environmental-mediated predictor of later development of psychopathic traits [91]. To this point, it would be interesting to explore to what extent psychopathic traits in parents may led to parenting practices that exhibit greater levels of harshness or psychological control (as well as reduced parental support and involvement) which, in turn, will influence the development of overall psychopathic and more specific CU traits [92, 93]. Contrastingly, if the parenting seems somewhat “normal,” that is free of harshness, harshness does not affect the development of GM or DI then research will need to further explore if there are certain parenting practices that lead to those traits or if the traits are present regardless of the environment. This may mean that the traits come about naturally and are independent of parenting practices. It overall highlights the importance of include environmental factors in the study of psychopathic traits, in order to accurately depict their role in the development of this personality profile and, therefore, their potential usefulness for prevention.

## Theoretical and Practical Implications

The current study has several theoretical and practical implications. First, although much more research is needed, the current results advance the properties of the PSCD-SV as a promising tool to accurately assess psychopathic personality from a multidimensional perspective including CD, which aligns with previous studies on the measure [12, 15]. Also in support of its psychometric properties, the PSCD-SV was a reliable measure and correlated in the expected manner with external correlates. These results gain relevance since the need of short measures for measuring personality traits has been increasingly recognized [52], particularly when large-scale surveys, to measure multiple constructs, are used. As Luo et al. [15] recommended, the PSCD-SV might be useful in studies where time limited due to other taxing aspects of a research study. For instance, in longitudinal designs where participants have to repeatedly complete large batteries or neuroscience studies where the imaging takes considerable time. The thirteen-item version cuts the time to complete the measure to just over half the time the original version takes to complete by reducing the measure by 10 items. However, where possible and where psychopathy is the chief study variable, we continue to recommend the full 24-item version of the PSCD. Regardless of the version used, the PSCD appears to a practical tool and it allows for the assessment of psychopathic traits in conjunction with CD. This is also signifies that the PSCD may be useful in clinical settings where CD and personality traits are of relevance to better understand the personality features that may be associated with youth with conduct problems.

Second, the current study may have implications for nomenclature and the DSM-5 [94] and the ICD-11 [95] classification of CD. Notwithstanding the advances and contributions from the CU-based approach, the evidence from this study indicates that the wider construct of psychopathy as well as its component parts could be quite useful for understanding youth with conduct problems. The findings from the current study correspond with other research collected so far across multiple studies conducted in childhood and adolescence which supports the multidimensionality of the psychopathy construct at early developmental stages [7, 11, 12, 15]. In this regard, the findings from the current study lend further support to the suggestion that all psychopathy dimensions, and not only CU traits, should be included as potential specifiers for CD in developmental models and diagnostic classification systems [9, 23, 64].

Third, from a theoretical perspective research aimed at disentangling the heterogeneity of conduct problems, with a wider set of psychopathic traits, may help with interventions. This is one of the few studies that examined the contribution of parenting practices in predicting psychopathic traits, taking into account all its dimensions rather than only CU traits. While this study demonstrates the psychometric properties of the PSCD-SV, it also shows how the PSCD, and measures that consider the broader construct, may have important implications for developmental models aimed at better understanding the mechanisms involved in the development of psychopathy and related behavioral problems, but also for prevention and intervention purposes. Hence, the identification, assessment, and management of those factors able to enhance, maintain, or reduce psychopathy over time should gain more relevance. In this regard, parenting practices should be included in prevention and intervention strategies specifically tailored to the specific needs of children with psychopathic traits. In fact, some promising results from the applied context have shown that focusing on improving parental practices has clinical value not only in reducing problematic behavior in children with high psychopathic traits, but also in favoring a significant reduction in all interpersonal, affective, and behavioral features of psychopathic personality [28, 96], although much more work is needed on this topic.

Notwithstanding these contributions, one should bear in mind that these results should be interpreted with caution since the label of psychopathy, as well as psychopathic traits or psychopathic personality, could be pejorative and stigmatizing, particularly when applied at early developmental stages. Yet, when research purposes are prioritized, the study of psychopathic personality in children and adolescents gain relevance since it allows to better understand the development of a disorder with its roots in early childhood [97] and, as was previously mentioned, to improve prevention and intervention strategies that will help to restrain more serious and pervasive patterns of CD and antisocial behavior [9]. Further research should be conducted on the label in conjunction with CD as has been done in the past [98, 99]. Even though a component part (e.g., callous-unemotional traits) have been purported to be less stigmatizing, these terms may also require testing.

## Limitations and Future Lines of Research

This study is not exempt of some limitations that must be taken under consideration. First, relying just on self-reports may have raised the possibility that observed effects were partially due to shared method variance, which means that magnitude and significance of relationships might be overestimated because they were reported by the same informant. Although previous research has indicated the validity and reliability of adolescent self-reports when reporting parenting practices and behavioral problems [100], the use of additional multi-informant approaches is particularly encouraged in future research to confirm relationships and accuracy in estimations. Second, given the nature of the current sample (i.e., community based), the mean levels for psychopathic traits might not be representative of high levels, suggesting the need for replication analyses in high-risk and clinic-referred samples. Third, because psychopathic traits were assessed in the third wave of the study, we were not able to explore test–retest validity, longitudinal stability, and potential interplays between psychopathic traits and parenting practices. Finally, although differences across gender groups could be expected in the observed results, sample size did not allow to accurately examining the results based on gender, a gap that should be particularly filled in future research.

## Summary

The current study examined the PSCD-SV in a sample of adolescents. The PSCD was designed to examine the dimensions of psychopathy in conjunction with CD. Unique to the PSCD is its ability to help clinicians and researchers answer many questions and concerns pertaining to the connections between psychopathy and CD. Other features of the PSCD are the inclusion of an ODD item as well as the focus on daring traits as opposed to impulsivity. These features make the PSCD different from other psychopathy scales (e.g., APSD, PCL-YV, YPI, YPI-S), making it a potential consideration for researcher and clinicians who are concerned with these various relations or who are interested in an alternate measure of psychopathy. Although additional research is needed, the findings from the current study indicated that this new measure of psychopathy and CD had relatively good psychometric properties, including adequate scale homogeneity and external validity. It suggests that a short version of the measure may well be valuable in research that is aiming to examine psychopathic traits in relation to conduct problems in adolescent samples. This was the first time that the PSCD psychometric properties have been examined in relation to parenting variables, including parental support and psychological control, and simultaneously highlighted how the multicomponent model of psychopathy could be important for investigating the mechanisms (even if partially) that lead to the development of psychopathy. Such investigations are likely to help the field better determine the most appropriate interventions for those children and adolescents with elevated conduct problems and psychopathic traits.
